# miR-132 overexpression is associated with modulation in miR-21 expression and glioblastoma cell behavior

**DOI:** 10.1371/journal.pone.0352119

**Published:** 2026-07-10

**Authors:** Kimia Abdi, Hadi Bayat, Seyed Javad Mowla

**Affiliations:** 1 Department of Molecular Genetics, Faculty of Biological Sciences, Tarbiat Modares University, Tehran, Iran; 2 Biochemical Neuroendocrinology, Montreal Clinical Research Institute (IRCM), affiliated with the Division of Clinical and Translational Research, Faculty of Medicine and Health Sciences, McGill University, Montréal, Canada; Rutgers University, UNITED STATES OF AMERICA

## Abstract

Glioblastoma multiforme (GBM) is the most aggressive and lethal primary tumor of the central nervous system. MicroRNAs (miRNAs) are key post transcriptional regulators of gene expression, and increasing evidence suggests that miRNA relationships may contribute to regulatory complexity in cancer biology. In this study, we combined *in silico* analyses of ten independent miRNA expression datasets (TCGA and GEO) with functional validation in GBM cell models to investigate the association between miR-132 and miR-21 in GBM. Differential expression analysis consistently demonstrated significant overexpression of miR-21-5p and downregulation of miR-132-3p in GBM tissues compared with normal brain. These findings were validated in U87 and C6 GBM cell lines using qRT-PCR, confirming consistent dysregulation of both miRNAs in vitro (p < 0.05). Functional experiments demonstrated that miR-132 overexpression is associated with reduced miR-21 expression and increased expression of established miR-21 target genes, including *BMPR2* and *BCL11B* at both mRNA and protein levels. These molecular changes were accompanied by reduced metabolic activity, impaired wound closure, and increased apoptotic cell death in both U87 and C6 GBM models. Collectively, these findings support a strong functional association between miR-132 expression and miR-21 related regulatory networks and phenotypic changes in GBM. However, the present study does not provide evidence for a direct physical or fundamental interaction between miR-132 and miR-21. Further mechanistic studies, including rescue experiments and direct binding assays, are required to clarify the underlying regulatory mechanisms.

## Introduction

Glioblastoma multiforme (GBM), arising from astrocytic cells, is the most aggressive and lethal primary brain tumor, accounting for approximately 50% of gliomas, with an average survival of about 14 months [1,2]. Despite multimodal treatment strategies, including surgical resection followed by chemotherapy and radiotherapy, therapeutic outcomes remain poor due to extensive tumor infiltration and pronounced genetic heterogeneity [3]. Therefore, identifying novel molecular targets that contribute to GBM progression remains a critical demand [4]. Non-coding RNAs (ncRNAs), particularly microRNAs (miRNAs), play essential roles in cancer biology by regulating gene expression at the post transcriptional level through mRNA degradation or translational repression [5]. Dysregulation of miRNAs is a hallmark of tumor progression and has led to the development of therapeutic strategies aimed at modulating miRNA activity [3,6]. Beyond canonical miRNA–mRNA interactions, emerging evidence suggests that miRNAs may participate in wider regulatory networks that indirectly influence the expression or activity of other miRNAs; however, the biological relevance and mechanistic core of such miRNA:miRNA relationships remain largely unexplored in GBM [7,8]. These interactions have been proposed to occur through three types: i) direct base-pairing between two miRs, ii) indirect interactions through shared mRNA targets, and iii) global network effects [7]. However, the biological relevance and mechanistic basis of such interactions in GBM remain largely hypothetical and insufficiently validated. Among dysregulated miRNAs in GBM, miR-21 is one of the most consistently upregulated oncomiRs and is known to promote tumor progression by suppressing multiple tumor suppressor genes, including *BMPR2* and *BCL11B* [9,10]. In contrast, miR-132 exhibits context dependent regulatory functions and has been implicated in tumor suppressive roles across multiple malignancies [11]. Dysregulation of miR-132 expression has been observed in several malignancies [12]. Notably, although miR-21 and miR-132 display inverse expression patterns in GBM, it remains unclear whether this relationship reflects a functional regulatory axis, an indirect network association, or independent deregulation events. Their potential coordinated effects on downstream targets and GBM associated cellular phenotypes have not been systematically investigated. In this study, we investigated the association between miR-132 and miR-21 expression and their downstream regulatory and phenotypic effects in GBM by integrating *in silico* analyses with functional validation in GBM cell. Specifically, we aimed to evaluate whether miR-132 is associated with changes in miR-21 expression, miR-21 target gene regulation, and key GBM related cellular phenotypes.

## Materials and methods

### *In silico* analysis

Ten independent miRNA expression datasets (one TCGA cohort and nine GEO datasets), comprising GBM and non-tumoral brain tissue samples, were retrieved using the TCGAbiolinks package (RRID: SCR_017683) as summarized in Table S1 in S1 File. Inclusion criteria required availability of raw or processed miRNA expression data with clearly defined control and GBM sample groups. Datasets lacking appropriate controls, incomplete metadata, or incompatible platforms were excluded from further analysis. To ensure comparability across sequencing and microarray platforms, each dataset was first processed independently according to its original preprocessing pipeline. Subsequently, TCGA miRNA-seq and GEO microarray datasets were normalized within each dataset prior to integration. Expression matrices were then harmonized using log2 transformation followed by Z-score standardization applied independently to each dataset. Batch effects arising from differences in platforms and study origins were corrected using the ComBat algorithm implemented in the sva package (RRID: SCR_012836). The effectiveness of batch correction was evaluated using principal component analysis (PCA) performed before and after normalization, which demonstrated a reduction in dataset-specific clustering while preserving the biological separation between GBM and normal brain samples (Supplementary Figure S1 in S1 File). Differential expression analysis was conducted using the LIMMA package (RRID: SCR_010943), and p-values were adjusted for multiple testing using the Benjamini–Hochberg method to control the false discovery rate (FDR).

### Plasmid construction

The precursor sequence of human miR-132 was synthesized by GeneScript (Piscataway, NJ, USA) and subsequently cloned into the mammalian expression vector pcDNA3.1(+) (Invitrogen, Carlsbad, CA, USA; Cat# V79020) to generate a miR-132 overexpression construct. Briefly, both the miR-132 insert and the pcDNA3.1(+) backbone were digested with BamHI (Thermo Fisher Scientific, Waltham, MA, USA; Cat# ER0055) and EcoRI (Thermo Fisher Scientific; Cat# ER0521), followed by ligation using T4 DNA ligase according to the manufacturer’s instructions.

The construct was designated as pmir-132. An empty pcDNA3.1(+) vector processed under the same conditions was used as a negative control. Correct insertion and orientation of the miR-132 sequence were confirmed by restriction enzyme analysis and Sanger sequencing. All oligonucleotide and primer sequences used in this study are listed in Table S2 in S1 File.

### Cell culture and electroporation

Human GBM cell line U87 (ATCC Cat# HTB-14, RRID: CVCL_0022) and rat glioma cell line C6 (ATCC Cat# CCL-107, RRID: CVCL_0194) were cultured in DMEM/F12 medium (Gibco, Cat# 12500096) supplemented with 10% fetal bovine serum (FBS; Gibco, Cat# A4736401) and 1% penicillin-streptomycin (Gibco, Cat# 10378016) under standard cell culture conditions (37°C, 5% CO₂). For electroporation, 2.5 × 10⁶ cells were resuspended in 500 µL PBS (Thermo Fisher Scientific) and mixed with 3 µg of miR-132 overexpression plasmid (pcDNA3.1(+)-miR-132; pmir-132). Cells were electroporated using a Gene Pulser Xcell system (Bio-Rad, Hercules, CA, USA) in 0.4 cm cuvettes (VWR, Radnor, PA, USA; Cat# 732−2924) under optimized conditions (250 V, 950 µF). Following electroporation, cells were kept on ice for 10 min, then gently transferred into 1 mL complete culture medium and plated in 6-well plates for recovery and further incubation for 48 h. Cells transfected with empty pcDNA3.1(+) vector served as the negative control. Transfection efficiency was evaluated 48h post-electroporation by quantifying mature miR-132 expression using qRT-PCR.

### RNA extraction, cDNA synthesis, and qRT-PCR

Total RNA was extracted using TRIzol reagent (Invitrogen, Carlsbad, CA, USA; Cat# 15596026) and treated with DNase I (Thermo Fisher Scientific; Cat# EN0521). Mature miR-132 and miR-21 expression levels were quantified by SYBR Green qRT-PCR (BioFact, Daejeon, South Korea; Cat# DQ385) using 5S rRNA as reference. mRNA levels (ACTB for C6 and HPRT for U87) were normalized using the 2^(-ΔΔCt) method. Data were analyzed using StepOne Software v2.3 (Applied Biosystems, RRID: SCR_014281).

### Wound-healing assay

U87/C6 cells (6 × 10⁴ per well in 12-well plates) were electroporated and subjected to scratch formation 6 h post-transfection using a 200 µL pipette tip. Cells were imaged at 0, 24, and 48 h using an inverted microscope. Gap closure was quantified using ImageJ software (NIH, Bethesda, MD, USA; RRID: SCR_003070). This assay provides a measure of gap closure that reflects a combined outcome of cell migration and proliferation dependent repopulation; therefore, it does not allow discrimination between these two processes and was interpreted beside proliferation related assays.

### MTT assay

Electroporated U87/C6 cells were seeded into 96-well plates. At 24, 48, and 72 h post-transfection, MTT solution (Sigma-Aldrich; Cat# M2003; 5 mg/mL, 10 µL per well) was added and incubated for 4 h. The medium was then removed, and formazan crystals were dissolved in 100 µL DMSO (Merck, Darmstadt, Germany; Cat# D8418). Absorbance was measured at 570 nm using an ELx800 microplate reader (BioTek, Winooski, VT, USA; Cat# ELx800). This assay measures cellular metabolic activity as an indirect indicator of cell viability and growth related cellular state, and may not directly reflect absolute cell number or proliferation rate; therefore, results were interpreted in conjunction with apoptosis and wound-healing assays to infer functional changes in cell behavior.

### Apoptosis assay

Electroporated U87/C6 cells (6 × 10⁴ per well in 12-well plates) were stained 48 h post-transfection using the Annexin V-FLUOS Staining Kit (Roche, Basel, Switzerland; Cat# 11988549001) according to the manufacturer’s instructions. Cells were incubated for 20 min at room temperature in the dark and subsequently analyzed using a FACSCalibur flow cytometer (BD Biosciences, Franklin Lakes, NJ, USA). Data analysis was performed using FlowJo v10 (RRID: SCR_008520).

This assay was used for quantification of apoptotic and viability related cell populations based on Annexin V/propidium iodide staining.

### Western blot assay

Total protein was extracted using RIPA buffer (Cell Signaling Technology, Danvers, MA, USA; Cat# 9806) and protease inhibitors (Roche, Cat# 11836153001). Proteins were separated by SDS-PAGE, transferred to PVDF membranes (Millipore, Burlington, MA, USA; Cat# IPVH00010), blocked (5% skim milk/TBST, 2.5 h, RT), and probed overnight (4°C) with: anti-ACTB (43 kDa; 1:1000; Santa Cruz Biotechnology, Dallas, TX, USA; Cat# sc-47778; RRID: AB_10920400), anti-BCL11B (116 kDa; 1:1000; Cat# sc-365988; RRID: AB_2833259), and anti-BMPR2 (115 kDa; 1:1000; Cat# sc-393304; RRID: AB_2938568). Blots were visualized using an ECL kit (Beyotime, Shanghai, China; Cat# P0018S), imaged (Canon EOS 60D), and quantified using CLIQS with ImageJ densitometry (normalized to ACTB). Protein expression levels of selected genes were evaluated.

### Statistical analyses

Data are presented as mean ± standard deviation from at least three independent biological replicates. Statistical significance was assessed using unpaired Student’s t-test or one-way ANOVA where appropriate. Multiple comparisons in in silico analyses were corrected using the Benjamini–Hochberg method to control false discovery rate. A p < 0.05 was considered statistically significant.

## Results

### Potential interaction between miR-132 and miR-21 and *in silico* analysis of miR-132 expression

**Fig 1:**
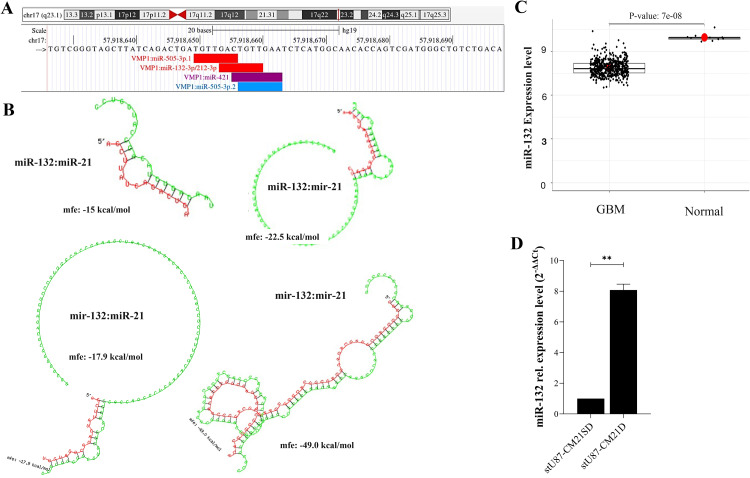
Combined computational and experimental analysis of the relationship between miR-132 and miR-21 in GBM. **(A)** UCSC Genome Browser-based miRNA interaction prediction indicating potential sequence complementarity between miR-132 and miR-21, identifying candidate pairing regions. **(B)** RNAhybrid analysis illustrating predicted base pairing configurations between miR-132-3p and miR-21, with corresponding minimum free energy (mfe) values, indicating thermodynamically promising duplex formation under computational conditions. **(C)** Integrated expression analysis of miR-132 across ten independent datasets (one TCGA cohort and nine GEO datasets; GBM n = 670, normal n = 60). Differential expression analysis using LIMMA with empirical Bayes moderation demonstrates a consistent and significant downregulation of miR-132 in GBM compared with normal brain tissues. **(D)** Relative expression level analysis of miR-132 expression in stable U87 GBM cells expressing a circular miR-21 sponge construct (stU87-CM21D) versus scrambled control cells (stU87-CM21SD). Inhibition of miR-21 activity was associated with a significant increase in miR-132 expression (two-tailed unpaired t-test, p = 0.0015). Data are presented as mean ± SEM.

Using UCSC based miRNA interaction prediction tools, miR-132 was identified among several candidate miRNAs with potential sequence complementarity to miR-21 (Fig 1A). To further examine this *in silico* observation, RNAhybrid was applied to estimate the thermodynamic stability of the putative miRNA duplexes and to identify the most favorable base pairing configurations. The analysis revealed several possible interaction patterns between miR-132 and miR-21, with minimum free energy values ranging from −15 to −49 kcal/mol, indicating that some predicted duplexes could form stable and energetically favorable interactions under computational conditions (Fig 1B). Nevertheless, these results should be interpreted cautiously, as bioinformatic predictions alone do not establish direct physical binding or functional interaction in cells. Therefore, the computational findings provide only a preliminary basis for subsequent experimental validation. To determine the expression pattern of miR-132 in GBM, we performed a meta-analysis of ten independent miRNA expression datasets (one TCGA cohort and nine GEO datasets), comprising GBM samples (n = 670) and non-tumoral brain tissues (n = 60). To ensure cross platform comparability, datasets were normalized and batch corrected prior to integration, and differential expression analysis was conducted using the LIMMA framework with empirical Bayes moderation. Across the integrated dataset, miR-132 expression was consistently reduced in GBM samples compared with normal brain tissue. This difference remained statistically significant after multiple testing correction (Benjamini–Hochberg adjusted p = 3.092 × 10 ⁻ ¹⁶), indicating a strong and reproducible downregulation pattern across independent cohorts. Visualization of the aggregated data (Fig 1C) further supports this observation, showing a clear separation between GBM and normal samples (p = 7 × 10 ⁻ ⁸). A total overview of differentially expressed miRNAs, including the position of miR-21 and miR-132 is provided as a volcano plot in Supplementary Figure S2 in S1 File, illustrating their expression patterns in GBM. Together, these results demonstrate a consistent and statistically strong downregulation of miR-132 in GBM across multiple independent datasets. Given that miR-21 is consistently overexpressed in GBM, we next examined whether modulation of miR-21 activity is associated with changes in miR-132 expression. To address this, we utilized a previously established stable U87 GBM cell model expressing a circular miR-21 sponge construct (stU87-CM21D), designed to sequester miR-21 molecules, and compared it with a scrambled control cell line (stU87-CM21SD). qRT-PCR analysis demonstrated a significant increase in miR-132 expression in stU87-CM21D -expressing cells compared with control cells (p = 0.0015, two-tailed unpaired t-test) (Fig 1D). The mean relative expression of miR-132 was markedly elevated in stU87-CM21D cells (mean = 8.08) compared with stU87-CM21SD controls (mean = 1.00). These findings indicate that suppression of miR-21 activity is associated with increased miR-132 expression, supporting a potential inverse relationship between these two miRNAs.

### Overexpression of miR-132 restores miR-21 target gene expression

**Fig 2:**
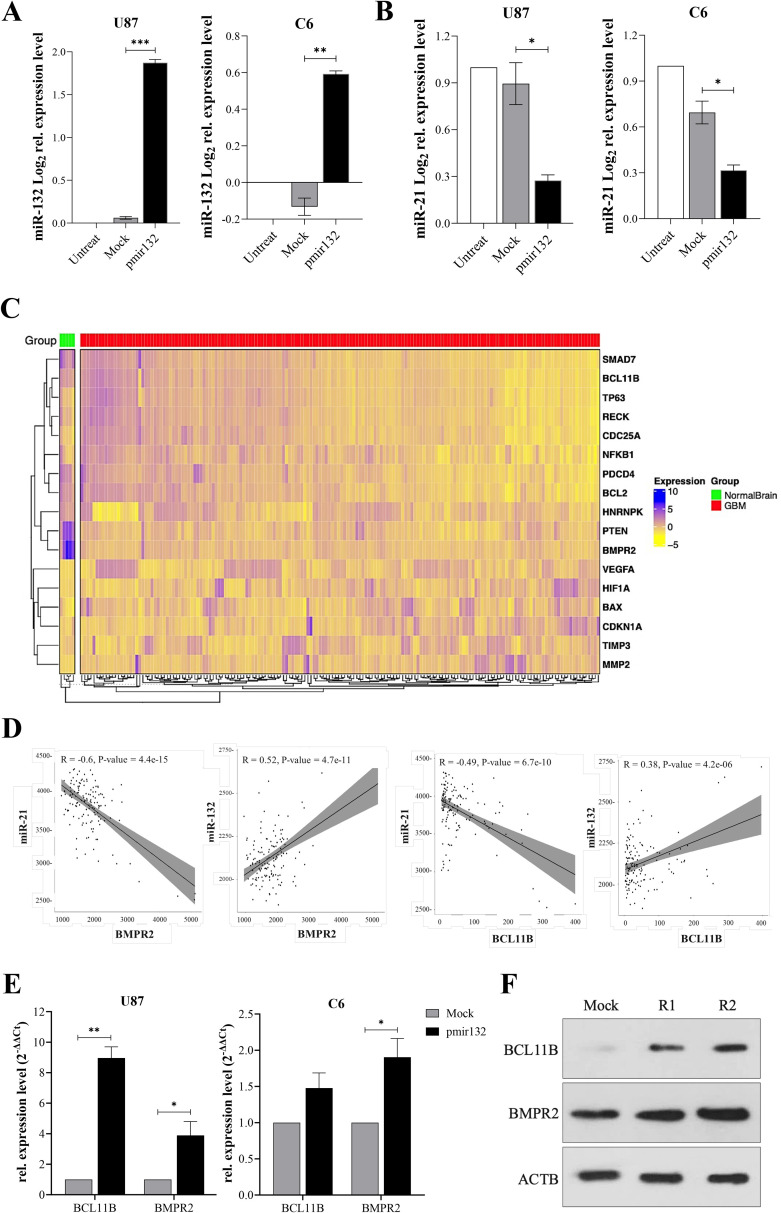
miR-132 associated modulation of miR-21 expression and downstream target genes in GBM. **(A)** qRT-PCR analysis of miR-132 expression in U87 and C6 cells following pmir132 transfection compared with mock controls. miR-132 expression was significantly increased in both U87 (p = 0.0003) and C6 (p = 0.0024) cells. **(B)** miR-21 expression was significantly reduced in pmir132 transfected U87 (p = 0.0242) and C6 (p = 0.0227) cells compared with controls. Data are shown as log₂ relative expression. **(C)** Heatmap of TCGA GBM datasets showing expression patterns of miR-21 target genes, including *BMPR2* and *BCL11B.* **(D)** Correlation analysis in TCGA GBM demonstrating inverse association between miR-21 and *BMPR2/BCL11B*, and positive association with miR-132. **(E)** qRT-PCR showed increased mRNA levels of *BMPR2* and *BCL11B* in pmir132 transfected cells (U87-*BCL11B*: 0.0042; U87-*BMPR2*: 0.0456; *C6-BCL11B*: 0.0841; *C6-BMPR2*: 0.0382; p < 0.05). **(F)**Western blot confirmed concordant protein upregulation with ACTB as loading control.

To evaluate the effect of miR-132 modulation in GBM, U87 and C6 cell lines were transfected with pmir132 expression vector, and miRNA expression levels were assessed by qRT-PCR. Cells transfected with a mock (empty) vector were used as control conditions. As shown in Fig 2A, miR-132 expression was significantly increased in pmir132 transfected cells compared with mock transfected controls in both U87 cells (p = 0.0003, unpaired two-tailed t-test) and C6 cells (p = 0.0024), confirming efficient overexpression of miR-132 across both GBM models. Subsequently, miR-21 expression was evaluated under miR-132 overexpression conditions (Fig 2B). Compared with mock transfected controls, miR-21 levels were significantly reduced in both U87 cells (p = 0.0242) and C6 cells (p = 0.0227) following miR-132 upregulation, with expression values reported as log₂ relative expression levels. Collectively, these results indicate that miR-132 overexpression is associated with a reduction in miR-21 expression in both GBM cell models. Importantly, no significant differences were observed between mock transfected and untreated control groups in either cell line, suggesting that the transfection procedure and vector backbone are unlikely to have independently influenced miR-21 or miR-132 expression levels. Accordingly, the observed changes in miRNA expression appear to be primarily associated with pmir132 mediated miR-132 overexpression. To investigate downstream regulatory targets of miR-21 in GBM, a comprehensive set of predicted and experimentally validated miR-21 target genes was first compiled based on miRNA–mRNA interaction databases (Fig 2C). To further support the relevance of these genes in GBM, transcriptomic expression patterns reported in GBM datasets were also evaluated, indicating consistent dysregulation of key miR-21associated targets in tumor tissues. As shown in Fig 2C, heatmap analysis of TCGA GBM datasets demonstrates a coordinated dysregulation pattern of established miR-21 target genes, including *BMPR2* and *BCL11B*, across tumor samples. To further examine the regulatory relationships at the expression level, *in silico* correlation analysis was performed using TCGA GBM datasets (Fig 2D). miR-21 expression exhibited a significant inverse correlation with *BMPR2* and *BCL11B*, consistent with their established role as miR-21 target genes. In contrast, miR-132 expression showed a positive correlation with both genes, suggesting a coordinated association between miR-132 expression and miR-21 downstream regulatory networks. Based on these results, *BMPR2* and *BCL11B* were selected as representative miR-21 target genes for subsequent experimental validation. To investigate the potential downstream consequences of miR-132 mediated modulation of miR-21 activity, we next examined the expression of two established miR-21 target genes, *BMPR2* and *BCL11B*, in GBM cell models. Experimental validation was performed in U87 and C6 cells following transfection with the pmir132 expression construct. As shown in Fig 2D, qRT-PCR analysis demonstrated a significant increase in relative mRNA expression levels of both *BCL11B* and *BMPR2* compared with mock transfected controls (p < 0.05 for all comparisons). The relative expression values (log2/ΔΔCt-based) showed increased expression in U87 cells for *BCL11B* (p = 0.0042) and *BMPR2* (p = 0.0456), as well as in C6 cells for *BCL11B* (p = 0.0841) and *BMPR2* (p = 0.0382), confirming consistent transcriptional upregulation across both models. Consistent with the transcript level findings, Western blot analysis was performed to evaluate protein expression of BCL11B and BMPR2 in U87 GBM cells following pmir132 transfection (Fig 2E). Representative immunoblot images demonstrated increased band intensity for both proteins in pmir132 transfected cells compared with mock transfected controls across biological replicates, while ACTB was used as a loading control to ensure equal protein loading (Fig 2F). The observed pattern was consistent in both cell lines, supporting the qRT-PCR results and indicating concordant regulation at the protein level. Collectively, these findings provide converging evidence that modulation of miR-132 is associated with a coordinated decrease in miR-21 expression and a corresponding increase in the expression of established miR-21 target genes at both transcript and protein levels in GBM cell models. This multi-level consistency suggests that miR-132 may participate in the regulation of miR-21associated signaling networks within GBM. Importantly, the observed expression patterns support a putative functional relationship within the miR-132:miR-2. However, the present study does not establish direct mechanistic interaction between the two miRNAs. In particular, assays such as luciferase reporter analysis or rescue-based experiments, which would be required to confirm direct regulatory causality, were not performed in this study. Therefore, the findings should be interpreted as indicative of an indirect regulatory association rather than a direct interaction. Despite this limitation, the integration of computational, transcriptomic, and functional cellular data provides a coherent framework suggesting a previously underexplored regulatory relationship between miR-132 and miR-21 in GBM.

### miR-132 modulates growth and motility related phenotypes in U87 GBM cells

**Fig 3:**
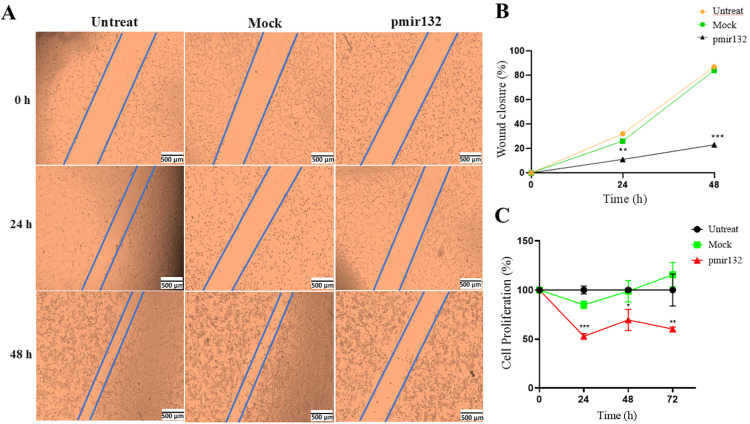
miR-132 associated effects on wound closure and cellular metabolic activity in GBM cells. **(A)** Representative images and quantitative analysis of wound-healing assay in U87 GBM cells following transfection with pmir132 compared with mock transfected and untreated control groups. miR-132 overexpression resulted in a significant delay in gap closure at 24 h and 48 h. **(B)** Quantification of wound closure in U87 cells, showing reduced percentage of gap closure in pmir132-transfected cells relative to both control groups. As scratch assays reflect a combined outcome of cell migration and proliferation-driven gap filling, the observed effect is interpreted as an integrated change in motility- and growth-associated cellular behavior rather than migration alone. **(C)** MTT assay in U87 and C6 GBM cells following pmir132 transfection. miR-132 overexpression significantly reduced cellular metabolic activity compared with mock-transfected controls, with a more pronounced effect observed at later time points, particularly at 72 h. Because MTT primarily reflects mitochondrial metabolic activity rather than absolute cell number, these results are interpreted as indicative of altered cellular viability and growth-associated metabolic state. Data are presented as mean ± SD/SEM, and statistical significance was assessed using unpaired two-tailed t-tests.

To investigate the functional consequences of miR-132 modulation in GBM cells, wound-healing and MTT assays were performed in U87 cells following transfection with pmir132, using untreated and mock transfected cells as control conditions. In the wound healing assay, pmir132 transfected cells exhibited a statistically significant delay in scratch closure compared with mock transfected controls at both 24 h and 48 h (24 h vs. 0 h: p = 0.0051; 48 h vs. 24 h: p = 0.0003) (Fig 3A, 3B). No significant differences were observed between mock transfected and untreated cells, indicating that the transfection procedure and vector backbone did not independently influence wound closure dynamics. However, given that wound healing assays do not distinguish between cell migration and proliferation, and considering the concurrent reduction in metabolic activity observed in the MTT assay, the delayed gap closure is interpreted as reflecting a combined effect on cell motility and proliferation dependent repopulation rather than migration specific changes. In parallel, MTT assays demonstrated a statistically significant reduction in cellular metabolic activity in pmir132 transfected cells compared with mock controls at 24 h (p = 0.0003), 48 h (p = 0.03), and 72 h (p = 0.001), while no significant differences were observed between mock transfected and untreated cells (Fig 3C). It is important to note that MTT primarily reflects mitochondrial metabolic activity rather than direct cell number; therefore, these results were interpreted as indicative of altered cellular metabolic state and viability rather than absolute proliferation rates. Taken together, these findings indicate that miR-132 overexpression is associated with statistically supported alterations in U87 GBM cell behavior, including delayed wound closure and reduced metabolic activity. Importantly, while these phenotypic changes are consistent with the observed modulation of miR-21 expression and its downstream targets, the present data support a functional association rather than a direct mechanistic relationship between miR-132 and miR-21.

### miR-132 modulates growth and motility related phenotypes in C6 GBM cells

**Fig 4:**
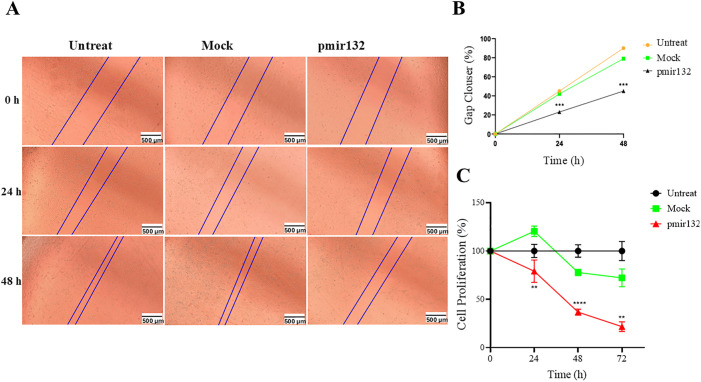
miR-132 associated effects on wound closure and metabolic activity in C6 GBM cells. **(A)** Representative images of wound-healing assay in C6 GBM cells following transfection with pmir132 compared with mock-transfected and untreated control groups, showing reduced gap closure in pmir132-transfected cells at 24 h and 48 h. **(B)** Quantitative analysis of scratch assay in C6 cells demonstrating a significant reduction in percentage of wound closure in pmir132-transfected cells relative to both control groups across time points. As wound-healing assays inherently reflect a composite of cell migration and proliferation-dependent gap filling, the observed effect is interpreted as an integrated change in motility- and growth-associated cellular behavior rather than a migration-specific phenotype. **(C)** MTT assay performed in C6 cells following pmir132 transfection. miR-132 overexpression significantly reduced cellular metabolic activity compared with mock controls at 24 h (p = 0.005), 48 h (p < 0.0001), and 72 h (p = 0.0011). No significant differences were observed between mock-transfected and untreated groups, indicating that the transfection procedure and vector backbone did not independently affect metabolic activity. Because MTT primarily reflects mitochondrial metabolic activity rather than direct cell number, results are interpreted as indicative of altered cellular viability and growth-associated metabolic state rather than absolute proliferation. Data are presented as mean ± SD/SEM, and statistical significance was assessed using unpaired two-tailed t-tests.

To evaluate whether the functional effects of miR-132 overexpression are conserved across GBM models, wound healing and MTT assays were performed in rat C6 glioma cells following transfection with pmir132, using untreated and mock transfected cells as control conditions. In the wound healing assay, pmir132 transfected C6 cells exhibited a statistically significant delay in gap closure compared with mock transfected controls at both 24 h and 48 h (24 h vs. 0 h: p = 0.0033; 48 h vs. 24 h: p = 0.0006) (Fig 4A). No significant differences were observed between mock transfected and untreated cells, indicating that the transfection procedure and vector backbone did not independently influence wound closure dynamics. Given that scratch assays reflect a combined outcome of cell migration and proliferation dependent gap filling, the observed reduction in gap closure is interpreted as an integrated effect on motility and growth associated cellular processes rather than migration alone. Quantitative analysis (Fig 4B) further confirmed a consistent and statistically supported reduction in closure percentage in pmir132 transfected C6 cells across both time points. Consistent with these findings, MTT assays (Fig 4C) demonstrated a statistically significant reduction in metabolic activity in pmir132 transfected C6 cells compared with mock controls at 24 h (p = 0.005), 48 h (p < 0.0001), and 72 h (p = 0.0011), while no significant differences were observed between mock transfected and untreated cells. As MTT primarily reflects mitochondrial metabolic activity rather than direct cell number, these results were interpreted as indicative of altered cellular viability and growth associated metabolic state rather than absolute proliferation rates. Taken together, these findings demonstrate that miR-132 overexpression is associated with statistically supported alterations in both wound closure dynamics and metabolic activity in C6 GBM cells, basically summarizing the phenotypic experiment observed in U87 cells. However, due to species specific differences between rat C6 and human GBM models, these results are interpreted as supportive of a conserved functional pattern rather than definitive evidence of translational relevance.

### miR-132 induces apoptotic cell death in U87 and C6 GBM cells

**Fig 5:**
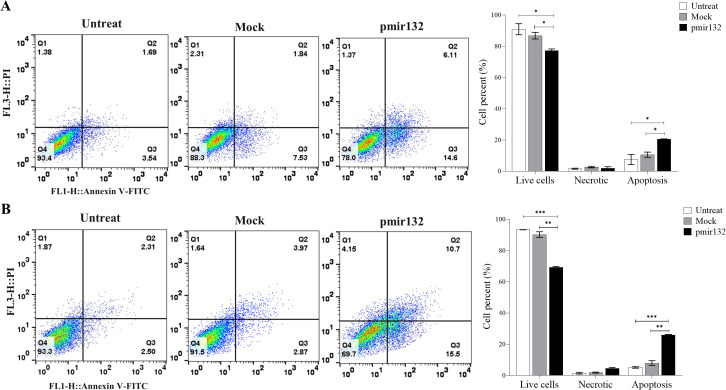
miR-132 induces apoptotic cell death in GBM cell models. **(A)** Annexin V/propidium iodide (PI) flow cytometry analysis of U87 GBM cells following transfection with pmir132 compared with mock-transfected controls. Representative dot plots and corresponding quantitative bar graphs illustrate the distribution of viable (Annexin V − /PI−), early apoptotic (Annexin V + /PI−), late apoptotic (Annexin V + /PI+), and necrotic (Annexin V − /PI+) cell populations. pmir132 transfection significantly increased total apoptotic cells (p = 0.0136) and reduced viable cells (p = 0.0286), while no significant change was observed in necrotic cells (p = 0.4667). **(B)** Annexin V/PI flow cytometry analysis of rat C6 glioma cells under the same experimental conditions. miR-132 overexpression significantly increased apoptotic cells (p = 0.0038) and reduced viable cells (p = 0.0041), with a modest but significant increase in necrotic cells (p = 0.0284) compared with mock controls. Data are presented as mean ± SD, and statistical significance was assessed using unpaired two-tailed t-tests.

To evaluate the effect of miR-132 modulation on cell survival, Annexin V/propidium iodide (PI) flow cytometry was performed in U87 GBM cells following transfection with pmir132, using mock transfected cells as control conditions. As shown in Fig 5A, representative flow cytometry dot plots together with corresponding quantitative bar graphs demonstrate the distribution of viable (Annexin V − /PI−), early apoptotic (Annexin V + /PI−), late apoptotic (Annexin V + /PI+), and necrotic (Annexin V − /PI+) cell populations in pmir132 transfected versus mock transfected cells. pmir132 transfection resulted in a marked increase in the apoptotic element compared with mock controls (p = 0.0136), indicating that miR-132 overexpression is associated with enhanced induction of programmed cell death in U87 cells. In parallel, a significant reduction in the proportion of viable (Annexin V − /PI−) cells was observed in the pmir132 group (p = 0.0286), reflecting an alteration in overall cell population dynamics away from survival toward apoptosis. In contrast, no significant difference was detected in the necrotic cell population between pmir132 and mock transfected cells (p = 0.4667), suggesting that membrane integrity was largely preserved and that the observed effects are not attributable to non-specific cytotoxicity. Quantitative summary of apoptotic and viability-related parameters is further illustrated in Fig 5A. To assess whether these effects are conserved across GBM models, Annexin V/PI analysis was also performed in rat C6 glioma cells following pmir132 transfection, using mock transfected cells as control conditions (Fig 5B). In C6 cells, miR-132 overexpression similarly led to a significant increase in apoptotic fraction compared with mock controls (p = 0.0038), accompanied by a significant reduction in viable cells (p = 0.0041). In addition, a ordinary but statistically significant increase in necrotic cells was observed in pmir132 transfected C6 cells (p = 0.0284), although the predominant effect remained associated with apoptotic cell populations. Collectively, these findings indicate that miR-132 overexpression consistently promotes apoptotic cell death in both human U87 and rat C6 GBM cell models, with a parallel reduction in viable cell parts. While U87 cells predominantly exhibit apoptosis without necrotic alteration, C6 cells show a minor increase in necrotic population, the overall pattern across both models supports a strong pro-apoptotic effect of miR-132. Importantly, these results further support a regulated induction of cell death rather than non-specific cytotoxicity, emphasizing the functional impact of miR-132 on GBM cell survival across distinct experimental schemes.

## Discussion

Our integrated *in silico* and experimental analyses consistently indicate that miR-132 overexpression is associated with decreased miR-21 levels and upregulation of known miR-21 targets such as *BMPR2* and *BCL11B* in GBM cell models. These molecular changes coincided with reduced metabolic activity, delayed wound closure, and increased apoptotic cell death in U87 and C6 cells. In agreement with previous meta-analyses, our analysis of ten independent GBM datasets confirmed that miR-21 is significantly upregulated and miR-132 downregulated in tumor tissues relative to normal brain. Moreover, in our cell based assays, inhibition of miR-21, using a sponge construct led to increased miR-132 expression, further suggesting an inverse relationship between these two miRNAs. Computational predictions (e.g. RNAhybrid) identified possible energetically promising pairings between miR-132 and miR-21, but such *in silico* findings require experimental validation and by themselves do not prove direct binding. Taken together, the data suggest a putative regulatory association between miR-132 and miR-21 networks in GBM rather than a confirmed miRNA:miRNA interaction. Importantly, we did not demonstrate a direct pivotal link between miR-132 and miR-21. The observed association could arise from indirect effects or extensive network interactions: for example, miR-132 might influence transcription factors or epigenetic regulators that in turn affect miR-21, or vice versa. Similar cross talk among miRNAs has been reported in other contexts, such as the proposed regulation of miR-21 by miR-122 in liver cells, though such mechanisms are not yet fully elucidated [13]. Clarifying the mechanism in GBM will require additional studies. Future experiments should include direct binding assay; for example, luciferase reporters containing the predicted miR-132 binding site on the miR-21 precursor, or pull-down assays to test whether miR-132 physically interacts with miR-21. Equally important are rescue experiments: for instance, co-transfecting a miR-21 mimic in miR-132 overexpressing cells or conversely inhibiting miR-21 would determine whether restoring miR-21 levels can reverse the effects on *BMPR2* and *BCL11B* expression and cellular phenotypes. Without such mechanistic validation, we interpret our findings as hypothesis generating evidence for an indirect association, not as proof of a novel miR-132:miR-21 regulatory interaction. Our study has several limitations that demand cautious interpretation. First, we used only two GBM models human U87 and rat C6 cells. Although both models showed consistent trends, they do not capture the heterogeneity of human GBM, and species-specific differences for example, in *BMPR2* and *BCL11B* regulation may limit the generalizability of our results. Validation in additional human GBM cell lines particularly patient derived or stem-like cell will be important to assess translational relevance. Second, the functional assays employed have inherent constraints. The wound-healing assay demonstrated slower gap closure with pmiR132, but this assay cannot distinguish effects on cell migration versus proliferation. Given that miR-132 also reduced metabolic viability (MTT assay), the delayed closure likely reflects combined effects on motility and growth. Future studies could use proliferation blocking agents (e.g., mitomycin C) during migration assays, or apply direct migration assays; such as trans-well migration or live cell imaging, to isolate motility effects. Similarly, the MTT assay measures metabolic activity rather than direct cell number; it was therefore interpreted as an indirect proxy for viability. Complementary assay; such as cell counting, EdU/BrdU incorporation, or colony formation would help confirm changes in proliferation and viability. Third, our findings are limited to in vitro models. In vivo studies (e.g., GBM xenografts or genetically engineered mouse models) will be necessary to evaluate whether miR-132 restoration can effectively counteract miR-21 driven oncogenesis in a physiological context. Despite these limitations, the data suggest that miR-132 may act to oppose miR-21 mediated oncogenic pathways. The observed upregulation of *BMPR2* and *BCL11B* with miR-132 overexpression is consistent with known tumor suppressive roles of these genes, and the attendant reduction in cell growth, migration and increase in apoptosis support a potential anti-tumor effect. From a therapeutic standpoint, these findings raise the possibility that miR-132 mimics could be used to indirectly suppress miR-21 associated networks in GBM. However, this remains speculative until in vivo efficacy and safety are demonstrated. In summary, our study identifies a consistent inverse association between miR-132 and miR-21 expression in GBM, with miR-132 overexpression linked to upregulation of miR-21 target genes and tumor-suppressive phenotypes. We emphasize that this relationship is currently correlative. Additional mechanistic work such as direct binding assays and rescue experiments is needed to establish causality and to elucidate the molecular basis of this putative miR-132:miR-21 regulatory axis. Nevertheless, these results provide a framework for future investigations into miRNA regulatory networks in GBM and suggest new avenues for targeting the miR-21 oncomiR.

## Supporting information

S1 File Supplemental information. This file contains Supplementary Tables S1–S2 and Supplementary Figures S1–S2.
(DOCX)

S1 Fig Graphical Abstract: Graphical summary of miR-132:miR-21 functional association in GBM. (JPEG)
