# Supplementary material for: miR-132 overexpression is associated with modulation in miR-21 expression and glioblastoma cell behavior
Source: PLoS One. 2026 Jul 10;21(7):e0352119. doi: 10.1371/journal.pone.0352119 (PMC13353934; doi:10.1371/journal.pone.0352119)
Supplement: S1 File — This file contains Supplementary Tables S1–S2 and Supplementary Figures S1–S2. (DOCX) [file pone.0352119.s001.docx]

**Supplemental Information**

**Table S1.** The list of datasets was retrieved from TCGA and GEO and used in this study. The number of normal and GBM subjects were indicated in each dataset.

| **Microarray study** | **Normal** | **GBM** | **Total** |
| --- | --- | --- | --- |
| TCGA-GBM | 10 | 496 | 506 |
| GSE103228 | 5 | 5 | 10 |
| GSE63319 | 4 | 14 | 18 |
| GSE61710 | 4 | 11 | 15 |
| GSE25631 | 5 | 82 | 87 |
| GSE42657 | 4 | 5 | 9 |
| GSE65626 | 3 | 3 | 6 |
| GSE90603 | 9 | 16 | 25 |
| GSE165937 | 4 | 9 | 13 |
| GSE158284 | 12 | 29 | 41 |
| Sum | 60 | 670 | 730 |

**Table S2.** The sequence of primers and oligonucleotides used in this study is listed.

| Primer Name |  | Sequence |
| --- | --- | --- |
| miR-132 | Forward | CGCTAACAGTCTACAGCCATG |
|  | Reverse | CCAGTGAGCAGAGTGACG |
| cir21-Divergent | Forward | ACGGTCGGGTCCAGATATTC |
|  | Reverse | CGTAGCTTATGTTCTGATGTTGATCT |
| cir21-convergent | Forward | CTCAACATCAGAACATAAGCTAGGC |
|  | Reverse | CCACACTCTACTCGACAGATACG |
| Scr21-Divergent | Forward | ACGGTCGGGTCCAGATATTC |
|  | Reverse | CGTGTTATTTAGTGGTCGCATTATCT |
| Scr21-Covergent | Forward | CTAATGCGACCACTAAATAACAGGC |
|  | Reverse | CCACACTCTACTCGACAGATACG |
| miR-21 | Forward | CCGGCCTAGCTTATCAGACTG |
|  | Reverse | AGTGCAGGGTCCGAGGTA |
| h5srRNA | Forward | GTCTACGGCCATACCACCCTG |
|  | Reverse | AAAGCCTACAGCACCCGGTAT |
| r5srRNA | Forward | TCTCGTCTGATCTCGGAAGC |
|  | Reverse | AGCCTACAGCACCCGGTATT |
| BMPR-11 | Forward | CTTGCCGTCTTGCTCATTC |
|  | Reverse | AGTCTATTTCCAGTCAGCCTC |
| HPRT | Forward | GCGTCGTGATTAGTGATGATGAA |
|  | Reverse | AGACGTTCAGTCCTGTCCTGTCCATAA |
| BCL11B | Forward | CTCCCTTTGGATGCCAGTGTCA |
|  | Reverse | GGCTCCAGGTAGATGCGGAAG |
| ACTB | Forward | AGCACAGAGCCTCGCCTT |
|  | Reverse | CATCATCCATGGTGAGCTGG |
| Gapdh | Forward | TGTGACTTCAACAGCAACTCCCAT |
|  | Reverse | CTCTCTTGCTCTCAGTATCCTTGC |
| mir132 | GGATCCTGACTCGCCGCCCCCGCCTCCTTCTGCTCCGCGTCCCCAGCCCGCGGCTCGGGGCGCGGCGTGGCGCGGCGCGTGGGCGTGCTGCGGGGCGACCATGGCTGTAGACTGTTACCTCCAGTTCCCACAGTAACAATCGAAAGCCACGGTTGCCCTGGAGACGCGGGGGCGGGGCGCGCGGACGGCGCCGGCGCGGGGCGGGCTGACGTCAGGGCCGCGGGGGGGCGGGCGGGGCGGCGGGGAATTC | |


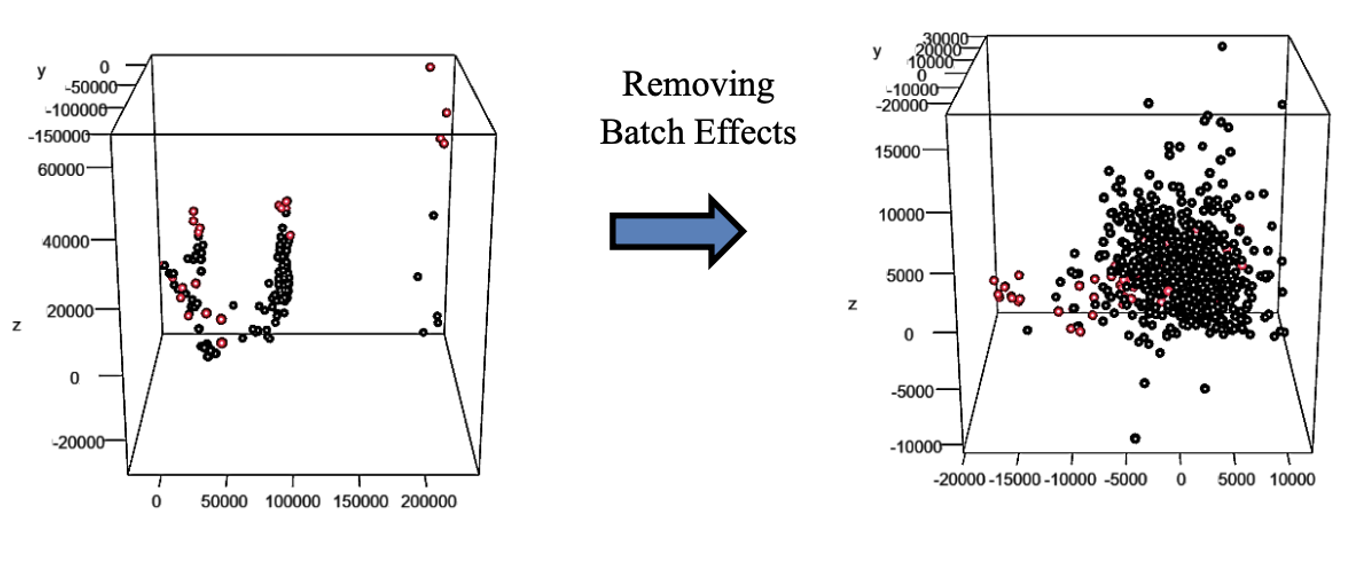


**Figure S1. Evaluation of batch effect correction.**

Principal component analysis (PCA) was performed before and after normalization and ComBat correction. Batch-associated clustering was reduced after correction, while separation between GBM and normal samples was preserved, indicating effective removal of technical variation.


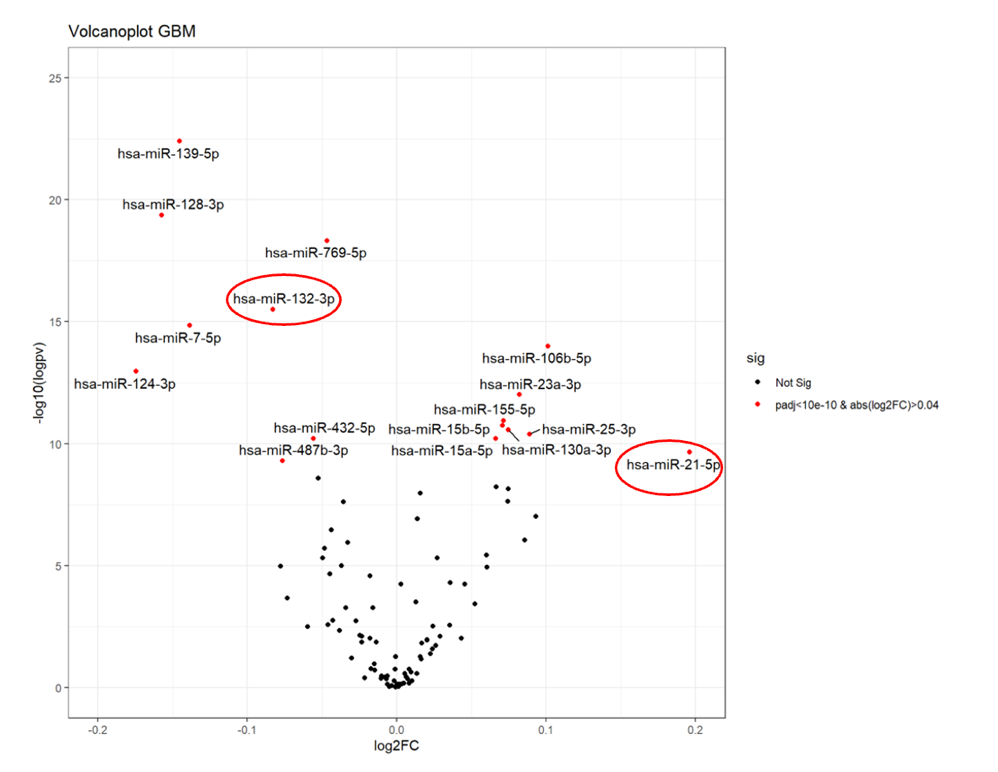


**Figure S2. Global differential expression profile of miRNAs in GBM.**
Volcano plot showing differentially expressed miRNAs between GBM and normal brain samples. The relative positions of miR-21 and miR-132 are highlighted, illustrating their distinct expression patterns within the overall distribution.
