# Supplementary figures and images for: miR-132 overexpression is associated with modulation in miR-21 expression and glioblastoma cell behavior
Source: PLoS One. 2026 Jul 10;21(7):e0352119. doi: 10.1371/journal.pone.0352119 (PMC13353934; doi:10.1371/journal.pone.0352119)

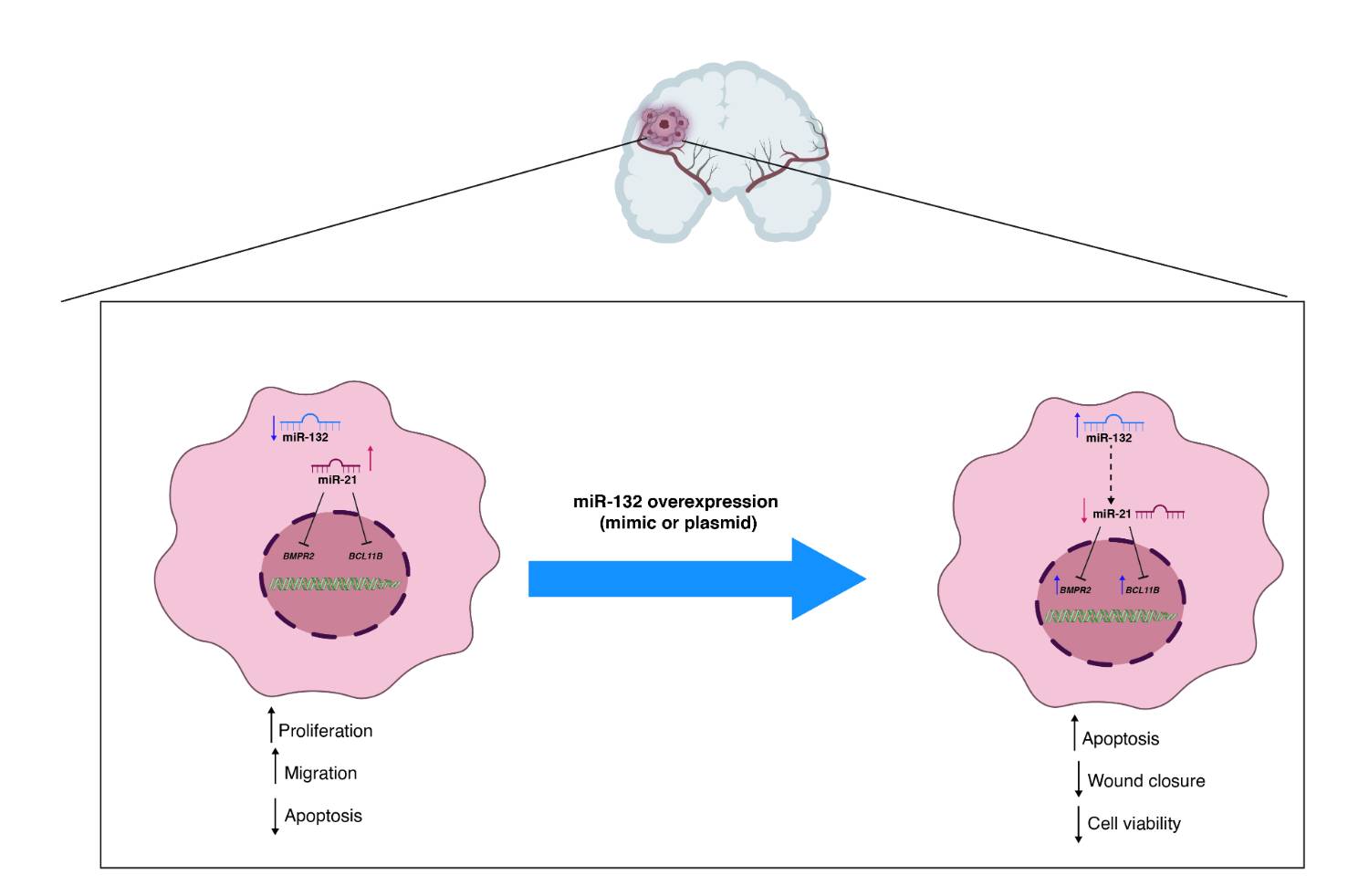

Supplement: S1 Fig — (JPEG) [file pone.0352119.s002.jpeg]
